# Editorial: E3 ubiquitin ligases: From structure to physiology to therapeutics, Volume II

**DOI:** 10.3389/fphys.2022.1038793

**Published:** 2022-09-27

**Authors:** Julien D. F. Licchesi, Heike Laman, Fumiyo Ikeda, Fleur M. Ferguson, Victor M. Bolanos-Garcia

**Affiliations:** ^1^ Department of Biology and Biochemistry, University of Bath, Bath, United Kingdom; ^2^ Department of Pathology, University of Cambridge, Cambridge, United Kingdom; ^3^ Graduate School of Frontier Biosciences, Osaka University, Osaka, Japan; ^4^ Skaggs School of Pharmacy and Pharmaceutical Sciences, University of California San Diego, San Diego, CA, United States; ^5^ Department of Chemistry and Biochemistry, University of California San Diego, San Diego, CA, United States; ^6^ Department of Biological and Medical Sciences, Oxford Brookes University, Oxford, England

**Keywords:** cullin-RING E3 ubiquitin ligase (CRL), *Dictyostelium discoideum*, E3 ubiquitin ligases, ligase-substrate degron recognition, SCF (Skp1-Cullin-F-box protein), stress granule (SG), cdc20, Cancer

Protein ubiquitination is a class of posttranslational modification present in eukaryotic cells that involves the addition of a ubiquitin (Ub) moiety to induce alteration in protein stability, function and subcellular localisation. It affects multiple cellular processes and is critical for timely protein degradation and signal transduction. This is a tightly regulated process that requires the concerted action of three enzymes. First, following Ub activation by ATP, Ub its covalent attached to an E1 Ub-activating enzyme, then transferred to an E2 ubiquitin conjugating enzyme, and subsequently transfer from the E2 enzyme to an E3 ubiquitin ligase, which is responsible for attaching the ubiquitin moiety to selected substrates with a high degree of specificity and selectivity.

A review by Okoye et al. discusses the manner in which E3 ubiquitin ligases engage their substrates through degradation signals known as “degrons”. Four examples of ligase:substrate degron recognition are outlined, including monovalency, where an E3 ligase’s single degron receptor binds a single degron in a substrate, and allovalency, where multiple copies of the same degron in a substrate can bind an E3 ligase’s single degron receptor. These different modes of ligase:substrate binding possess distinct affinities and specificities which likely bestows on these interactions unique kinetics. Illustrative examples of these various modes of binding for Nrf2/Keap1 (hetero-multivalent), MyD88/SPOP (mono-valent), Gli3/SPOP (homo-multivalent), and Sic1/Cdc4 (allovalent) are highlighted and focus our attention on the varied strategies that have co-evolved between substrates and ligases. The mitosis regulator Anaphase Promoting Complex/Cyclosome (APC/C) is presented as a unique example of a multi-subunit E3 ligase, employing multivalent interactions to promote the degradation of 100s of substrates containing various degrons (KEN, D-box, ABBA) recognized on distinct sites on its substrate docking subunits. The authors offer some useful insights gained from studying APC/C:substrate interactions for consideration when designing chimeric compounds for targeted proteolysis. Multivalency could be used to boost PROTAC efficacy, either by increasing the number of ligands on a PROTAC or alternatively, one could imagine, by enabling oligomers of recruited E3 ligases. The perspective offered by these authors encourages those in the ubiquitin field to take an over-arching view of the field to appreciate the molecular determinants that contribute to successful protein destruction.

Krause et al. discusses how ubiquitination of stress granule (SG) components contributes to their dynamics. SGs are biomolecular condensates mainly composed with RNAs and proteins and are localised in the cytosol. Their formation is induced by various types of cellular stress and also under pathogenic conditions. SG formation is governed by LLPS, which *in vitro* is regulated by diverse environmental factors including temperature, pH and osmolarity. Posttranslational modifications, including ubiquitination, play an important role in the dynamics of SG components. The authors focus on SG modifications by ubiquitin and ubiquitin-like molecules and discussed how these modifications regulate formation, dynamics and elimination of SGs. Since ubiquitin can form different linkage types of ubiquitin chains on substrates, they explain which types of chains are formed on which specific substrates and how they contribute to SG dynamics. There are several SG components and regulators known to be ubiquitinated. For example, TDP-43, FUS, VCP/p97, and G3BP are modified by different types of ubiquitin chains. Because linkage types of ubiquitin chains, ubiquitin ligases and deubiquitinases that regulate SGs remain largely unknown, the authors conclude that discoveries on these enzymes and ubiquitination types should be an important endeavour to exploit therapeutic strategies for diseases linked to aberrant SGs.

APC/C works in conjunction with CDC20 to mediate anaphase onset and mitosis exit. While yeast and animals only encode one copy of CDC20, in *Arabidopsis thaliana*, six isoforms have been reported, although their function and mode of regulation are currently unknown. Cosma et al. set out to compare the domain organisation all six *A. thaliana* CDC20 (AtCDC20) isoforms and show that a WD40 7-blade beta-propeller domain and the C-terminal I/T motif are fully conserved amongst isoforms and in human CDC20. Given the WD40 domain of human CDC20 mediates binding to APC/C during mitosis, it is likely that all AtCDC20 isoforms perform a similar function in plants. The authors used a combination of biochemical and biophysical assays to show the WD40 domain is globular, monomeric and stable at a broad range of pH. The authors then highlighted differences in the short linear motif (SLiMs) which are located towards the N-terminal portion of CDC20 and put forward possible explanations for how these might contribute differently to individual AtCDC20 isoforms. When compared to human CDC20, *A. thaliana* isoforms were found to lack a putative MAD1 binding site, a CRY-box degron and phosphorylation sites for BUB1 and PLK1 kinases. Overall, this work provides novel insights on putative new functions of *A. thaliana* CDC20 isoforms which could have broader implications for our understanding of eukaryotic cell division.

*Dictyostelium discoideum* is a model organism that has been studied for nearly a century. *D. discoideum* 24 h life cycle comprises unicellular and multicellular phases, enabling the study of fundamental cellular and developmental processes such as cell-cell communication and adhesion as well as cell differentiation. These biological processes are highly relevant to human physiology in normal and disease conditions. Kim et al. discuss the roles of the Skp1-Cullin-F-box (SCF) complex, a member of the cullin-RING E3 ubiquitin ligase (CRL) superfamily in the regulation of *D. discoideum* growth and development and of neddylation in the regulation of cell migration and chemotaxis. Kim et al. also discuss the extent of conserved functions of cullins across animal and plant species and the importance of neddylation, a post-translational modification consisting of the removal of NEDD8 (Neural precursor cell expressed developmentally down-regulated protein 8) from target proteins through the actions of the COP9 signalosome, to control the activity of the SCF complex. Because diverse studies in *D. discoideum* and humans indicate that neddylation plays an important regulatory role in chemotaxis, *D. discoideum* has great potential to improve our understanding of the molecular mechanisms by which CRLs regulate chemotaxis in humans. Moreover, also in humans, abnormalities in neddylation are associated with autoimmune diseases, neurodegeneration, and cancer. It will be interesting to see to what extent *D. discoideum* can be exploited as a model system to study these malignancies.

In summary, this Research Topic highlights some of the most significant discoveries in the structure to physiology to therapeutics understanding of E3 ubiquitin ligases.

